# Functional Toxicogenomic Profiling Expands Insight into Modulators of Formaldehyde Toxicity in Yeast

**DOI:** 10.3389/fgene.2016.00200

**Published:** 2016-11-17

**Authors:** Matthew North, Brandon D. Gaytán, Carlos Romero, Vanessa Y. De La Rosa, Alex Loguinov, Martyn T. Smith, Luoping Zhang, Chris D. Vulpe

**Affiliations:** ^1^Department of Nutritional Science and Toxicology, University of CaliforniaBerkeley, CA, USA; ^2^Division of Environmental Health Sciences, School of Public Health, University of CaliforniaBerkeley, CA, USA

**Keywords:** formaldehyde, yeast, functional genomics, alternative models

## Abstract

Formaldehyde (FA) is a commercially important chemical with numerous and diverse uses. Accordingly, occupational and environmental exposure to FA is prevalent worldwide. Various adverse effects, including nasopharyngeal, sinonasal, and lymphohematopoietic cancers, have been linked to FA exposure, prompting designation of FA as a human carcinogen by U.S. and international scientific entities. Although the mechanism(s) of FA toxicity have been well studied, additional insight is needed in regard to the genetic requirements for FA tolerance. In this study, a functional toxicogenomics approach was utilized in the model eukaryotic yeast *Saccharomyces cerevisiae* to identify genes and cellular processes modulating the cellular toxicity of FA. Our results demonstrate mutant strains deficient in multiple DNA repair pathways–including homologous recombination, single strand annealing, and postreplication repair–were sensitive to FA, indicating FA may cause various forms of DNA damage in yeast. The SKI complex and its associated factors, which regulate mRNA degradation by the exosome, were also required for FA tolerance, suggesting FA may have unappreciated effects on RNA stability. Furthermore, various strains involved in osmoregulation and stress response were sensitive to FA. Together, our results are generally consistent with FA-mediated damage to both DNA and RNA. Considering DNA repair and RNA degradation pathways are evolutionarily conserved from yeast to humans, mechanisms of FA toxicity identified in yeast may be relevant to human disease and genetic susceptibility.

## Introduction

Extensive industrial and commercial uses of formaldehyde (FA) results in both high production volumes and consequent exposure potential (National Toxicology Program (NTP), 2010). FA is utilized to produce industrial resins and adhesives and can serve as a disinfectant or preservative (IARC, 2012a). In addition to its industrial production, combustion, smoking of cigarettes, and secondary photochemical reactions of hydrocarbon pollutants can generate FA. Moreover, FA is produced endogenously through metabolic processes in humans and animals.

FA's ubiquity results in broad potential for occupational and environmental exposure (IARC, 2012a). Workplace exposure to FA has been linked to nasopharyngeal, sinonasal, and lymphohematopoietic cancers, leading both the International Agency for Research on Cancer (IARC) and the U.S. National Toxicology Program (NTP) to classify FA as a human carcinogen (National Toxicology Program (NTP), 2010; IARC, 2012a). *In vitro*, FA at high levels can be cytotoxic, and lower exposure can induce DNA damage, expressed as DNA adducts and DNA-protein crosslinks, as well as chromosome changes, expressed as chromosomal aberrations, sister chromatid exchanges, and micronuclei (National Toxicology Program (NTP), 2010; Zhang et al., 2010a). *In vivo*, high doses of FA can cause necrosis, and increased levels of DNA-FA adducts have been identified after exogenous inhalation to FA in experimental animals (Moeller et al., 2011; Yu et al., 2015). In humans, increased DNA-FA and protein-FA adduct levels have been reported in smokers and exposed workers (Pala et al., 2008; Wang et al., 2009; Bono et al., 2012). Further, hematotoxicity and elevated leukemia-specific chromosome changes in myeloid blood progenitor cells were found among Chinese workers exposed to high levels of FA (Zhang et al., 2010b; Lan et al., 2015).

Although the genotoxicity of FA is well established, the biological mechanisms, genes, and pathways underlying FA toxicity and susceptibility in humans—for both cancer and non-cancer adverse health effects—are not well understood. To examine human genetic susceptibility to FA (i.e., gene-environment interactions), genome-wide association studies and candidate gene association studies are needed; however, these approaches require large exposed and control populations or some knowledge of mechanisms of toxicity (McHale et al., 2014). An alternative approach utilizes unbiased functional screens in the eukaryotic budding yeast *Saccharomyces cerevisiae* to identify candidate human FA susceptibility genes. *S. cerevisiae* represents an attractive model for understanding the cellular mechanisms of FA toxicity and/or susceptibility. Fundamental cellular, metabolic, and signaling processes are conserved between yeast and more complex organisms. Human homologs or functional orthologs exist for a considerable portion of the yeast genome (Steinmetz et al., 2002), and many essential yeast genes can be substituted with their human orthologs (Kachroo et al., 2015). Furthermore, abundant genetic and physical interaction data, bioinformatic resources, and genetic screening tools increase the utility of yeast in studying FA toxicity and susceptibility.

Chemical-genetic profiling (or functional profiling), in which collections of yeast deletion mutants are screened in parallel for altered growth in a substance of interest, has provided important insight into pharmaceutical and toxicant mechanisms of action (Giaever et al., 2002; reviewed by North and Vulpe, 2010). Functional studies in yeast can guide further experimentation in more complex organisms, as various genetic requirements for chemical tolerance identified in yeast have been confirmed in models such zebrafish or human cell lines (reviewed by Gaytán and Vulpe, 2014).

In this study, a genome-wide functional screen was performed with the *S. cerevisiae* non-essential deletion collection to identify the genetic requirements for yeast FA tolerance. We identified components of multiple DNA repair pathways as required for FA tolerance, suggesting DNA damage contributes to FA toxicity in yeast. Unexpectedly, we also identified a genetic requirement for multiple subunits of SKI, a protein complex that regulates mRNA degradation by the exosome. Many of the yeast genes identified in this study have functional human orthologs that may similarly modulate FA toxicity or susceptibility in humans.

## Materials and methods

### Yeast strains and culture

Genome-wide screens and individual strain analyses were conducted using the set of BY4743 non-essential diploid yeast deletion strains (*MATa/MAT*α *his3*Δ*1/his3*Δ*1 leu2*Δ*0/leu2*Δ*0 lys2*Δ*0/LYS2 MET15/met15*Δ*0 ura3*Δ*0/ura3*Δ*0*, Invitrogen). Yeast cultures were grown at 30°C in liquid rich media (1% yeast extract, 2% peptone, 2% dextrose, YPD) with shaking at 200 rpm.

### Dose-finding and growth curve assays

Formaldehyde solutions were diluted from a 37% stock solution (Sigma-Aldrich, St. Louis, MO) immediately before use. Dose-finding and growth curve assays were performed as described (North et al., 2011). FA solutions were added to the desired final concentrations, with at least two technical replicates per dose. Area under the curve (AUC) data for each strain were derived from three independent biological replicates. Statistical significance between wild-type and mutant strains was calculated with Student's *t*-test.

### Functional profiling assays

Growth of the pooled deletion strains (4607 mutants in total), genomic DNA extraction, strain barcode amplification, Affymetrix TAG4 array hybridization, and differential strain sensitivity analysis (DSSA) were performed as described (Jo et al., 2009). Data files are available at the NCBI Gene Expression Omnibus (GEO) database with accession number GSE83398.

### Enrichment analyses

Strains designated as sensitive by DSSA were input into the Functional Specification (FunSpec) software tool, using a *p*-value cutoff of 0.01 and Bonferroni correction, to identify overrepresented Gene Ontology (GO) and MIPS (Munich Information Center for Protein Sequences) categories. Further enrichment analyses were conducted with the Cytoscape software tool by mapping 5G and 15G sensitive strain fitness data (*n* = 225) onto the BioGRID yeast interaction data. In cases where strains were identified as sensitive in both 5G and 15G DSSA analyses, 15G fitness data was used. The Cytoscape plugin jActiveModules was utilized to search for subnetworks enriched with strain fitness data, and the BiNGO plugin subsequently identified overrepresented GO Biological Processes.

## Results

### Functional profiling of the yeast genome identifies genes required for formaldehyde tolerance

**Figure 1:**
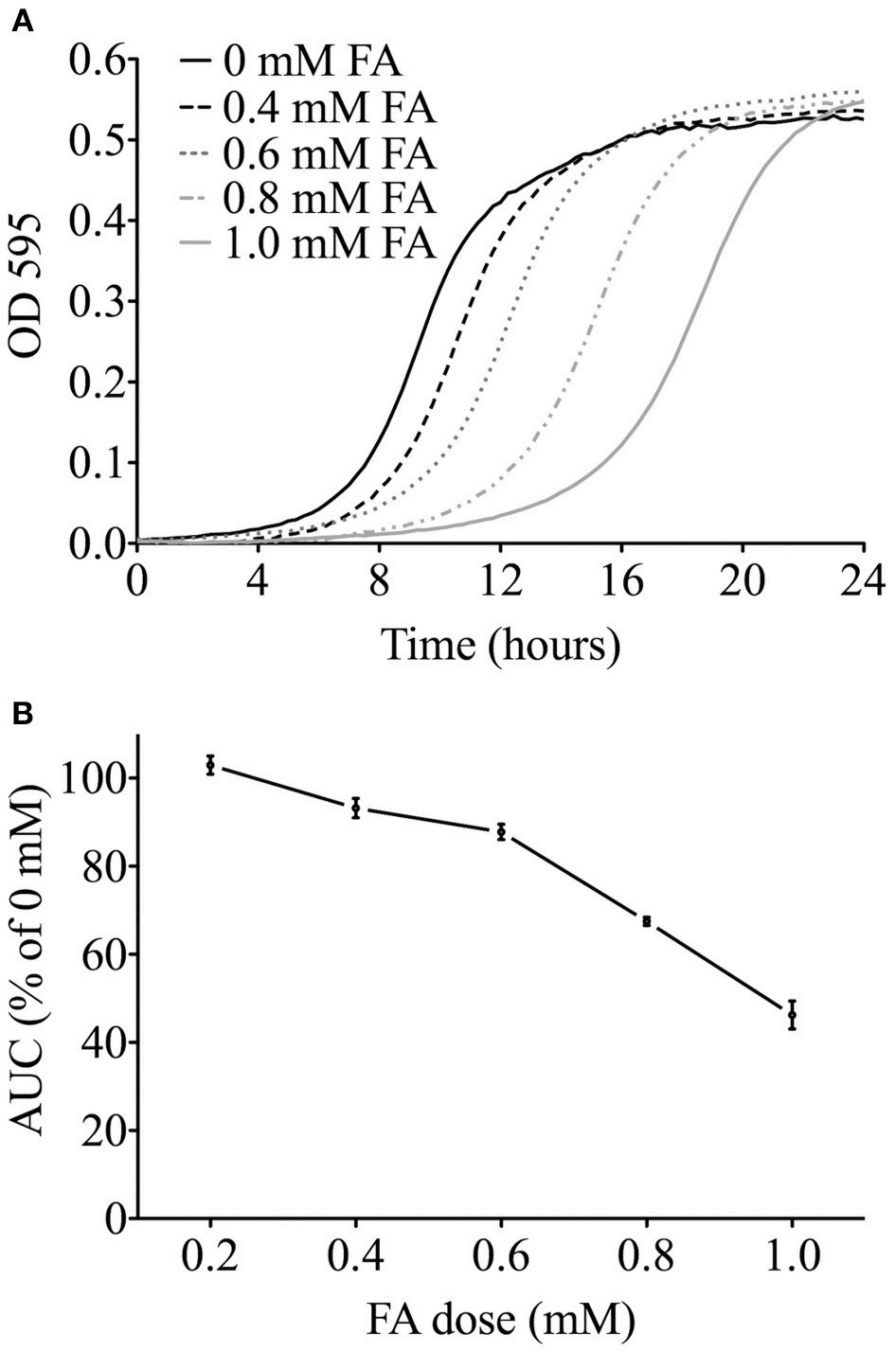
**Determining the FA IC_20_ for functional screens. (A)** Representative growth curves in YPD media for the wild-type BY4743 strain exposed to FA. For clarity, the 0.2 mM FA curve is not shown. **(B)** The area under the curve (AUC) was determined for each FA dose from three independent growth curve experiments, expressed as the mean and SE, and plotted as a percentage of the untreated control. The FA IC_20_ was calculated to be about 0.6 mM (600 μM).

A range of chemical doses and exposure times were utilized to identify candidate genes involved in modulating FA tolerance. We use the IC_20_–a concentration that inhibits growth of the wild type strain by 20%–as the highest dose to balance between sufficient toxicity to identify differential growth of mutant strains and non-specific toxicity that can be observed at higher doses in yeast functional screens to identify mutants that are more or less sensitive to a chemical stressor (Jo et al., 2009). To determine the FA IC_20_, growth curves were performed with wild-type yeast and increasing concentrations of FA (Figure 1A), with the IC_20_ calculated as 0.6 mM (Figure 1B). Non-essential deletion mutant pools were grown in 0.6 mM (IC_20_), 0.3 mM (50% IC_20_), and 0.15 mM (25% IC_20_) FA for either 5 or 15 generations (5G and 15G) to identify genes required for optimal growth in FA. DSSA revealed 225 strains were sensitive to one or more treatment with FA, with 149 strains common between the 5G and 15G treatments, and 32 strains sensitive to four or more of the six treatment conditions (Table S1). Strains were selected for follow-up growth curve confirmation assays based upon the results of overenrichment analyses described below. Strains sensitive to formaldehyde were the focus of this study.

### Overenrichment analyses reveal requirements for formaldehyde tolerance

**Figure 2:**
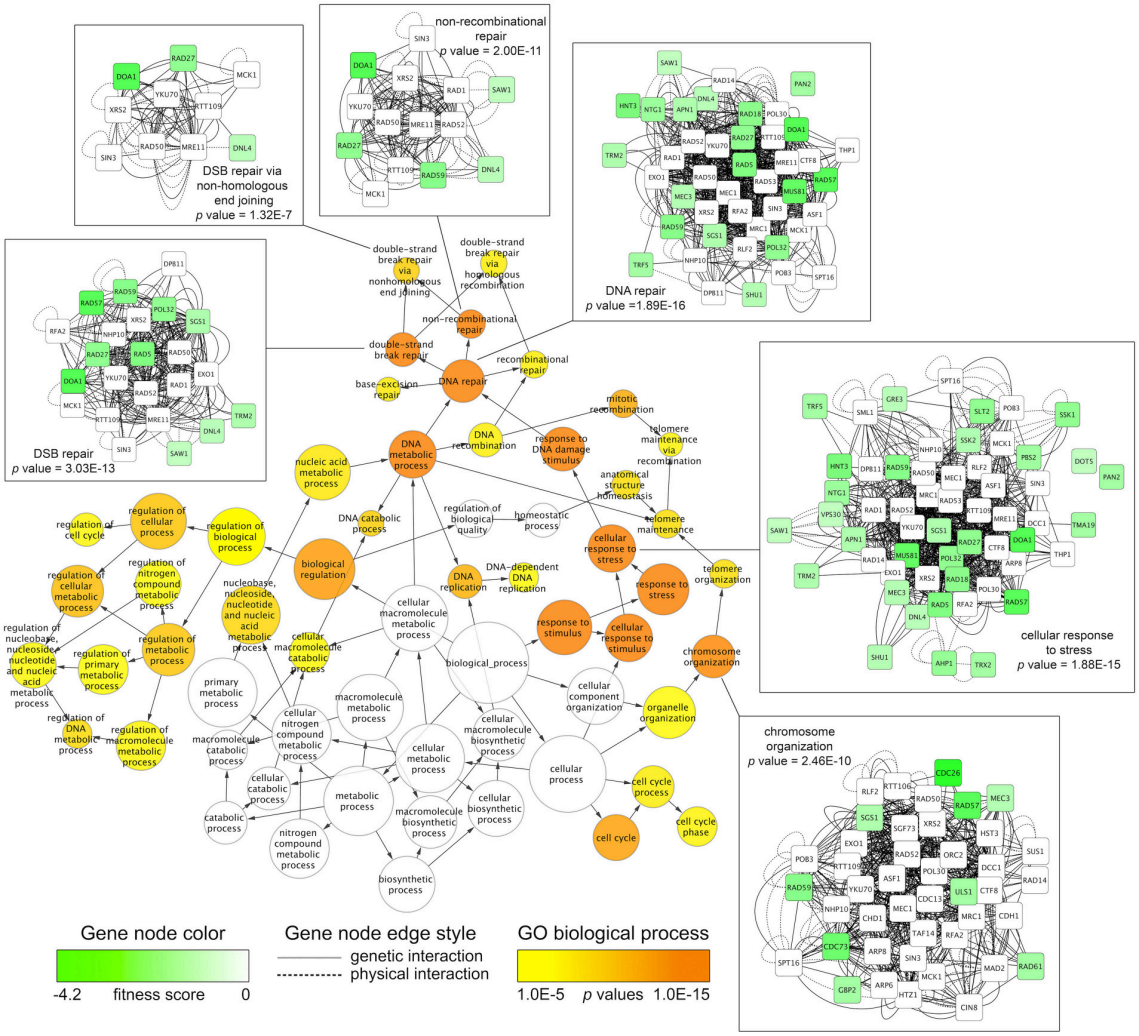
**Network mapping identifies biological processes required for FA tolerance**. The Cytoscape software tool was used to map fitness data for FA-sensitive strains onto the *S. cerevisiae* BioGRID interaction dataset (3.4.130 release). The jActiveModules plugin identified a genetic subnetwork (*n* = 204) enriched with fitness data, consisting of genetic and physical interactions between sensitive, non-sensitive, and essential genes. Using the subnetwork of 204 genes as input, the BiNGO plugin discovered significantly overrepresented Gene Ontology (GO) Biological Processes (*p*-value cutoff of 0.001). For clarity, GO categories at a *p*-value cutoff of 1E-5 (0.00001) are displayed, with all BiNGO output shown in Table S2. The BiNGO output node color (orange to yellow) and size correspond to *p*-values and number of genes, respectively. Edge arrows illustrate GO term hierarchy. Genetic subnetworks for selected GO categories are shown, where node color (green to white) corresponds to strain fitness score and edge indicates the type of interaction (physical/genetic) between the genes.

To discover overrepresented biological attributes within the 5G and 15G DSSA data, the FA sensitive strains (*n* = 225) were input into the FunSpec software tool. The analysis revealed four broad categories of genes required for FA tolerance, including those involved in DNA repair, RNA turnover (i.e., components of the SKI complex), osmoregulation, and the oxidative stress response (Table 1). Additional enrichment evaluations conducted with the Cytoscape network mapping software and the BiNGO plugin further demonstrated that various DNA repair genes, such as those involved in double strand break repair, along with those involved in stress response and chromosome organization, were needed for FA tolerance (Figure 2; Figure S1; Table S2).

**Table 1:** **Genes required for FA tolerance and their associated GO or MIPS categories**.

**GO Biological Process**	***p*-value**	**Genes identified**	**k^a^**	**f^b^**
Response to virus [GO:0009615]	1.31E-006	*SKI8, SLH1, SKI2, SKI3*	4	4
Nuclear-transcribed mRNA catabolic process, exonucleolytic, 3′–5′ [GO:0034427]	7.36E-004	*SKI8, SKI2, SKI7, SKI3*	4	13
DNA repair [GO:0006281]	1.38E-003	*NTG1, SAW1, RAD18, RAD59, RAD57, MUS81, RAD27, APN1, TRM2, RAD5, MEC3, SGS1, DNL4, HNT3, PHR1*	15	183
Nuclear-transcribed mRNA catabolic process, 3′-5′ exonucleolytic nonsense-mediated decay [GO:0070478]	2.20E-003	*SKI8, SKI2, SKI7, SKI3*	4	17
Response to DNA damage stimulus [GO:0006974]	2.85E-003	*NTG1, SAW1, RAD18, RAD59, RAD57, MUS81, RAD27, APN1, RAD5, MEC3, YIM1, SGS1, DNL4, HNT3, PHR1*	15	197
Nucleus organization [GO:0006997]	3.39E-003	*TOM1, GSP2*	2	3
Cellular monovalent inorganic cation homeostasis [GO:0030004]	3.39E-003	*NHX1, VHS3*	2	3
Telomere maintenance via recombination [GO:0000722]	3.40E-003	*RAD59, RAD57, MEC3, SGS1*	4	19
DNA recombination [GO:0006310]	3.50E-003	*RAD59, MUS81, SHU1, CDC73, SGS1, DNL4*	6	44
Cell redox homeostasis [GO:0045454]	3.62E-003	*GRX6, TRX2, DOT5, POR2, AHP1*	5	31
Nucleotide metabolic process [GO:0009117]	5.26E-003	*APA1, APA2, AMD1*	3	11
Response to singlet oxygen [GO:0000304]	6.63E-003	*SNQ2, SKN7*	2	4
Osmosensory signaling pathway via two-component system [GO:0007234]	6.63E-003	*SSK1, SSK2*	2	4
Nuclear-transcribed mRNA catabolic process, non-stop decay [GO:0070481]	6.98E-003	*SKI8, SKI2, SKI7, SKI3*	4	23
Cellular response to oxidative stress [GO:0034599]	7.43E-003	*NTG1, GRX6, TRX2, GRE3, DOT5, TMA19, AHP1*	7	67
CVT pathway [GO:0032258]	7.87E-003	*COG7, COG8, COG6, COG5, VPS30*	5	37
Nucleocytoplasmic transport [GO:0006913]	7.87E-003	*TOM1, NUP100, NUP188, NUP53, GSP2*	5	37
Intra-Golgi vesicle-mediated transport [GO:0006891]	8.16E-003	*COG7, COG8, COG6, COG5*	4	24
DNA metabolic process [GO:0006259]	8.67E-003	*RAD57, MUS81, MEC3*	3	13
Postreplication repair [GO:0006301]	8.67E-003	*PAN2, POL32, RAD5*	3	13
Response to drug [GO:0042493]	9.45E-003	*SNQ2, YKL075C, TDA5, IRC21*	4	25
**GO cellular component**	***p*****-value**	**Genes identified**	**k**^a^	**f**^b^
Ski complex [GO:0055087]	1.31E-006	*SKI8, SKI2, SKI7, SKI3*	4	4
Golgi transport complex [GO:0017119]	8.25E-005	*COG7, COG8, COG6, COG5*	4	8
Phosphopantothenoylcysteine decarboxylase complex [GO:0071513]	3.39E-003	*SIS2, VHS3*	2	3
Polysomal ribosome [GO:0042788]	6.63E-003	*SLH1, TMA46*	2	4
**MIPS functional classification**	***p*****-value**	**Genes identified**	**k**^a^	**f**^b^
DNA repair [10.01.05.01]	9.28E-005	*NTG1, RAD18, RAD59, RAD57, MUS81, ECM32, PAN2, POL32, RAD27, APN1, DOA1, RAD5, MEC3, DNL4, ULS1, PHR1*	16	159
Oxygen and radical detoxification [32.07.07]	5.24E-004	*TRX2, DOT5, SSK1, AHP1*	4	12
Electromagnetic waves stress response (e.g. UV, X-ray) [32.01.13]	1.16E-003	*RAD61, PHR1*	2	2
Detoxification by export [32.07.05]	3.39E-003	*QDR2, YRM1*	2	3
osmosensing and response [34.11.03.13]	6.19E-003	*SLT2, PBS2, SIS2, SSK1, SSK2*	5	35
RNA transport [20.01.21]	8.66E-003	*GBP2, NUP100, SRN2, NUP188, NUP53, TPM1, TEX1, GSP2*	8	86

Strains identified as sensitive to FA by DSSA (n = 225) were input into FunSpec and analyzed for overrepresented biological attributes.

a *Number of genes from category identified as sensitive to FA*.

b *Total number of genes in GO or MIPS category*.

### Yeast mutants deficient in formaldehyde metabolism are sensitive to formaldehyde

Both Sfa1p, the yeast FA dehydrogenase, and YJL068Cp, a S-formylglutathione hydrolase, have been implicated in formaldehyde metabolism, with deletions exhibiting sensitivity to FA (Wehner et al., 1993; Degrassi et al., 1999; de Graaf et al., 2009). As a positive control, growth curve assays were performed with *sfa1*Δ and *YJL068C*Δ mutants, with both demonstrating sensitivity to FA as compared to a wild-type control (Figure S2).

### SKI complex mutants are sensitive to formaldehyde

**Figure 3:**
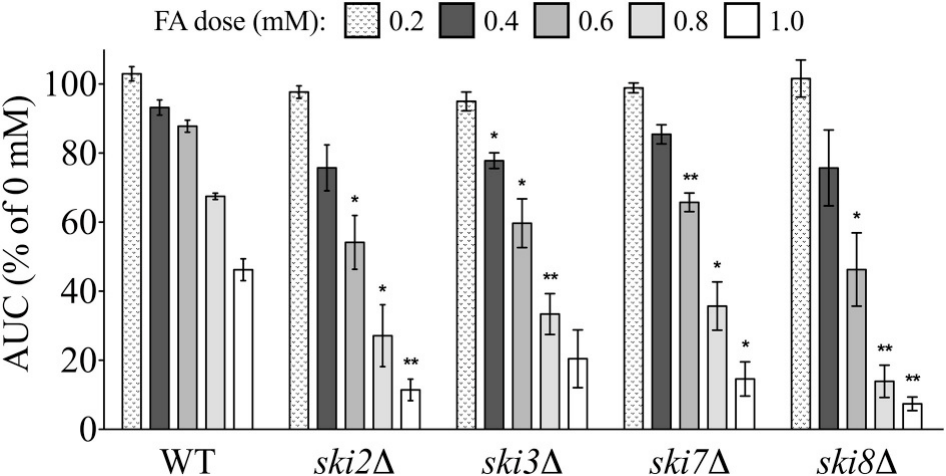
**SKI mutants are sensitive to FA**. The AUC was calculated for each strain after 24 h of exposure to the indicated concentrations of FA. Bars display mean AUC as a percentage of the untreated strain AUC with standard error (SE) for three independent replicates. Statistical significance between the wild-type and mutant strains was calculated with Student's *t*-test, where ^**^p < 0.01 and ^*^p < 0.05.

Overrepresentation analyses suggested the SKI complex–a mediator of RNA degradation by the exosome (Brown et al., 2000)–was required for FA tolerance. In *S. cerevisiae*, growth curves with increasing FA concentrations were determined for individual mutants lacking each of the three components of the SKI complex (*ski2*Δ, *ski3*Δ, and *ski8*Δ) as well as the protein that couples SKI to the exosome (*ski7*Δ). Growth curves for the mutants were compared to the wild-type strain, confirming that a fully functional SKI is required for growth in FA (Figure 3).

### Mutants defective in osmoregulation and stress response are sensitive to formaldehyde

**Figure 4:**
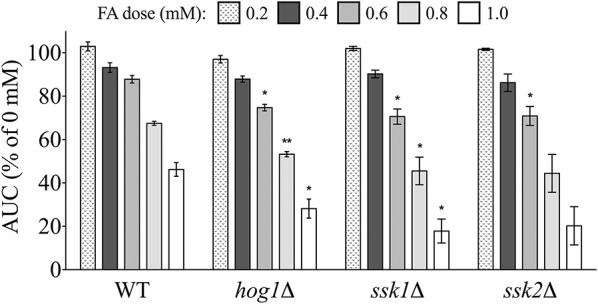
**Osmoregulation and stress response mutants are sensitive to FA**. The AUC was calculated for strains treated for 24 h with indicated concentrations of FA and expressed as a percentage of the AUC for the untreated strain. Bars show the mean and SE for three independent cultures. Statistical significance between the wild-type and mutant strains was calculated with Student's *t*-test, where ^**^p < 0.01 and ^*^p < 0.05.

Genes implicated in osmoregulation and stress response were also identified by enrichment analyses as required for tolerance to FA. We examined FA sensitivity in three mutants of the Hog1p pathway, which regulates expression of the previously described FA detoxification enzyme Sfa1p (Rep et al., 2001). The *hog1*Δ, *ssk1*Δ, and *ssk2*Δ individual mutants were exposed to increasing concentrations of FA and compared to the wild-type strain, with results indicating each of the three are sensitive to FA (Figure 4).

### Various DNA repair pathway mutants are sensitive to formaldehyde

Enrichment analyses identified DNA repair as one of the major biological processes needed for FA tolerance. Closer examination of the FunSpec and Cytoscape/BiNGO output demonstrated various categories of DNA repair mutants were sensitive to FA, including those deficient in single strand repair (i.e., base excision repair), double strand break (DSB) repair (i.e., via either homologous recombination [with a subcategory being break-induced replication], non-homologous end joining, or single-strand annealing), and post-replication repair (see Table 1, Figure 2, and Table S2).

#### Double strand break (DSB) repair mutants are sensitive to formaldehyde

**Figure 5:**
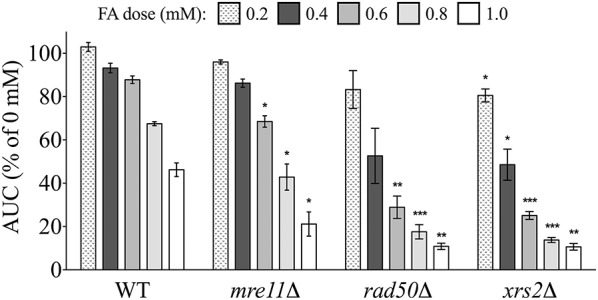
*****Mre11*** complex mutants are sensitive to FA**. The AUC was calculated for each strain treated for 24 h with various FA concentrations and expressed as a percentage of the AUC for the untreated strain. Bars show the mean and SE for three independent cultures. Statistical significance between the wild-type and deletion strains was calculated with Student's *t*-test, where ^***^p < 0.001, ^**^p < 0.01, and ^*^p < 0.05.

We first tested mutants lacking components of the trimeric *Mre11* (MRX) complex for sensitivity to FA, as this machinery is integral to processing of DSBs prior to repair by homologous recombination (HR) or non-homologous end joining (NHEJ) (reviewed by Krogh and Symington, 2004). Increased FA sensitivity of the *Mre11* complex strains *mre11*Δ, *rad50*Δ, and *xrs2*Δ indicates FA may cause DSBs (Figure 5), as *Mre11* complex mutants are hypersensitive to DNA damaging agents such as methyl methanesulfonate (MMS) (Chang et al., 2002).

#### Homologous recombination (HR) mutants are sensitive to formaldehyde

**Figure 6:**
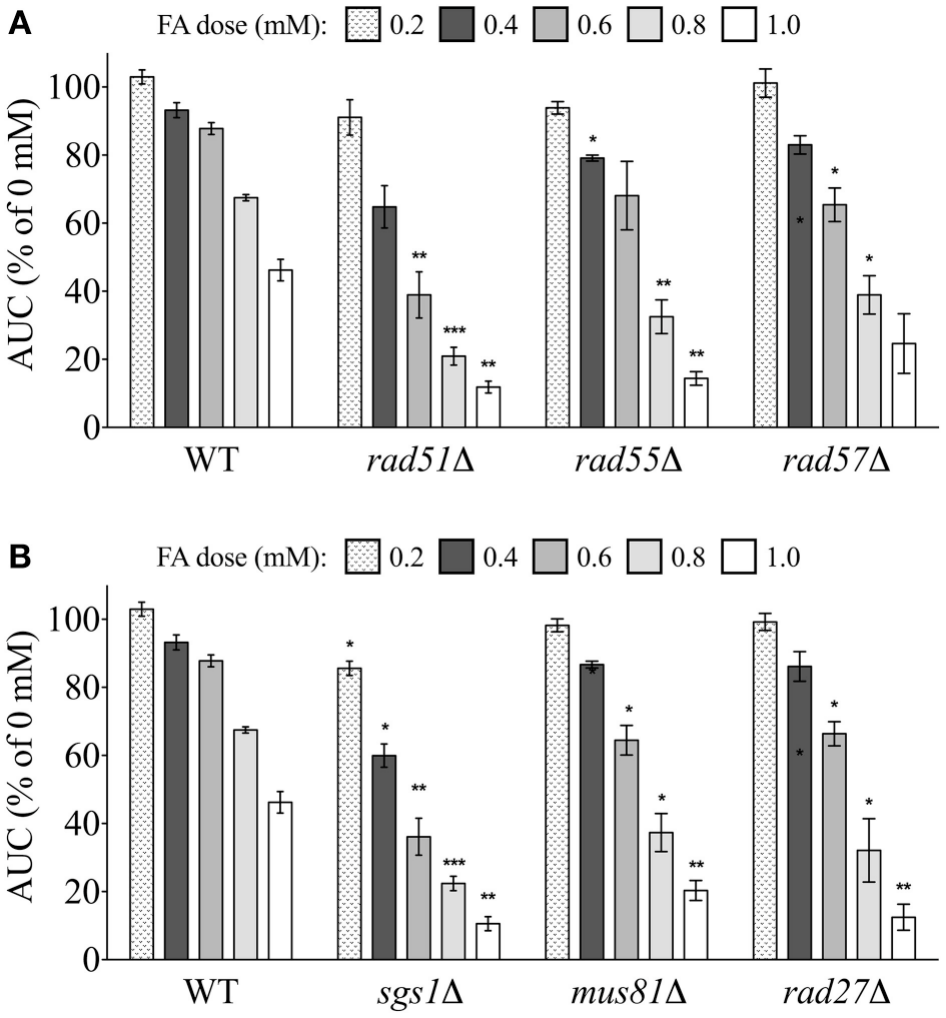
**Mutants defective in DSB repair via homologous recombination are sensitive to FA**. Strains were treated for 24 h with indicated FA concentrations and the AUC was calculated. Shown is the mean AUC as a percentage of the AUC for the untreated strain and SE for three independent cultures. Statistical significance between the wild-type and mutant strains was calculated with Student's *t*-test, where ^***^p < 0.001, ^**^p < 0.01, and ^*^p < 0.05.

We next examined the FA sensitivity of strains deficient in various aspects of DSB repair. DSBs can be repaired by different homologous recombination (HR) pathways. The strand invasion pathway of HR is capable of repairing DSB error-free and requires the Rad51p strand exchange protein to complex with single-stranded DNA (ssDNA) at a DSB (Namsaraev and Berg, 1997)–a process promoted by the Rad55p/Rad57p heterodimer. All three of these strand exchange mutants (*rad51*Δ, *rad55*Δ, *rad57*Δ) were sensitive to FA (Figure 6A). HR intermediates generated via strand invasion must be resolved by helicases (through dissolution) or structure-selective endonucleases (through endonucleolytic cleavage) (Heyer et al., 2010). The DNA helicase Sgs1p was an additional participant in the DSB repair via HR pathway needed for FA tolerance (Figure 6B). Furthermore, we found the *mus81*Δ strain—lacking an endonuclease—experienced growth defects in FA, and we also show that *RAD27*/Rad27p, which genetically and functionally interacts with *MUS81*/Mus81p (Thu et al., 2015), is needed for FA tolerance (Figure 6B).

#### Single strand annealing (SSA)-DSB repair mutants are sensitive to formaldehyde

**Figure 7:**
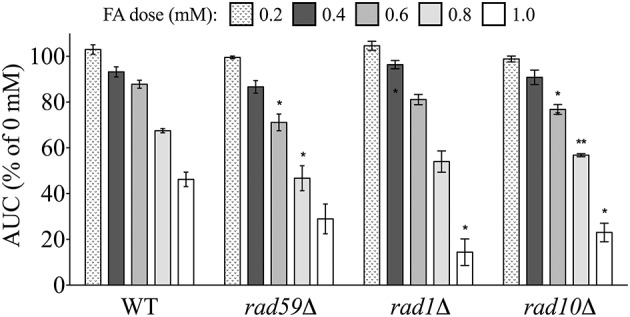
**Mutants defective in DSB repair via single strand annealing are sensitive to FA**. The AUC was determined for each strain after 24 h treatment with the indicated FA concentrations. Graph bars express AUC as a percentage of the AUC for the untreated strain and show the mean and SE for three independent experiments. Statistical significance between the wild-type and mutant strains was calculated with Student's *t*-test, where ^**^p < 0.01 and ^*^p < 0.05.

Another form of DSB repair that uses homologous sequences, single strand annealing (SSA), typically involves interactions between broken chromosome ends, and is considered an error-prone process (Frankenberg-Schwager et al., 2009). A strain deleted for Rad59p, which is linked to SSA and required for loading of Rad52p to DSBs (Davis and Symington, 2001), was sensitive to FA (Figure 7). Additionally, deletions of either Rad1p or Rad10p, which complex to help remove non-annealing tails during SSA as well as nucleotide excision repair (NER) (Tomkinson et al., 1993; Ivanov and Haber, 1995), produced FA sensitivity (Figure 7). Additional NER strains were not identified in the screen, and FA sensitivity was not exhibited in a strain lacking Saw1p, a protein that recruits Rad1p-Rad10p to SSA intermediates (data not shown). Therefore, our data suggest FA may induce DSBs that can be repaired by HR and SSA (and perhaps NER) processes, with HR serving as the preferred or dominant pathway over SSA (HR mutants being more sensitive to FA than SSA mutants).

#### Non-homologous end joining (NHEJ) mutants are not sensitive to formaldehyde

DSSA and enrichment analyses indicated that DSB repair via NHEJ (a non-recombinational error-prone process) was also required for FA tolerance (Figure 2; Figure S1; Tables S1, S2). The previously discussed *RAD27* (whose deletion results in FA sensitivity; see Figure 6B) has been additionally implicated in NHEJ (Tseng and Tomkinson, 2004), but surprisingly, deletion of major players in NHEJ pathways, including *DNL4*, a ligase required for NHEJ, and *YKU70*, a promoter of NHEJ, did not result in increased FA sensitivity (data not shown).

#### Post-replication repair (PRR) mutants are sensitive to formaldehyde

**Figure 8:**
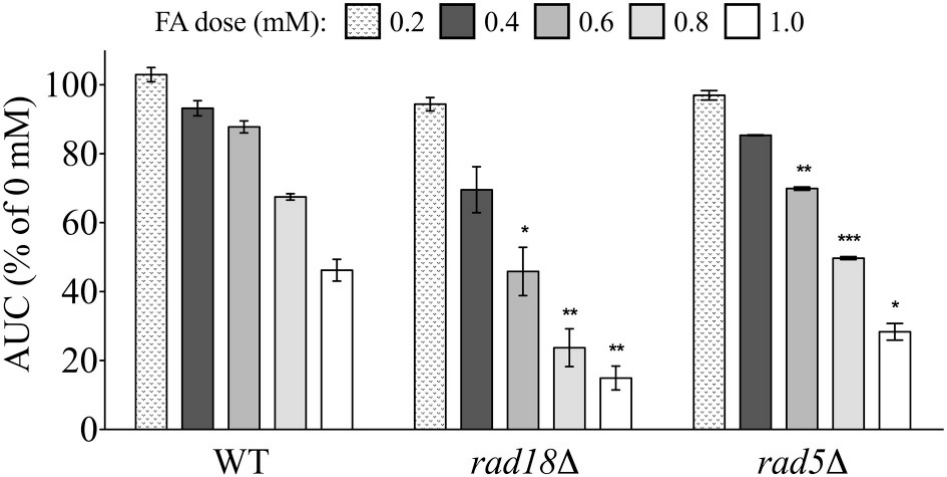
**Mutants defective in postreplication repair are sensitive to FA**. Strains were exposed for 24 h to various FA concentrations and AUCs were calculated. Bars express AUC as a percentage of the untreated strain AUC and display the mean and SE for three independent replicates. Statistical significance between the wild-type and mutant strains was calculated with Student's *t*-test, where ^***^p < 0.001, ^**^p < 0.01, and ^*^p < 0.05.

*PRR* was another category of DNA repair identified by enrichment analyses (Figure 2; Table S2; Figure S1) as required for FA tolerance. PRR encompasses multiple repair pathways that help resolve (1) mismatched pairs introduced by the replication machinery/polymerase during DNA synthesis or (2) lesions encountered during replication. In the latter case, error-free and error-prone mechanisms can bypass (tolerate) replication-blocking lesions in an effort to prevent fork stalling/collapse and the formation of DSBs at the expense of genomic integrity (reviewed by Prakash et al., 2005). We examined two PRR mutants lacking components involved in lesion bypass, *rad18*Δ and *rad5*Δ, for FA sensitivity. Rad18p is an E3 ubiquitin ligase that initiates lesion bypass via recruitment of low fidelity polymerases that do not remove damage, but instead continue replication past lesions and may thus introduce mutations (reviewed by Prakash et al., 2005). Meanwhile, Rad5p is a Rad6p/Rad18p dependent DNA helicase specializing in replication fork regression that promotes template switching to bypass damage in an error-free manner (Blastyák et al., 2007). Both *rad18*Δ and *rad5*Δ exhibited growth defects in FA (Figure 8), demonstrating that FA may induce DNA damage that impairs replication, with repair potentially occurring via both error-prone and error-free PRR pathways.

## Discussion

FA is an important high production volume chemical with considerable industrial and commercial applications. Exposure to FA has been associated with various nasal and blood cancers, prompting its classification as a human carcinogen (IARC, 2012a; National Toxicology Program (NTP), 2010). Despite evidence demonstrating FA's genotoxicity and additional non-cancer adverse effects, the mechanisms of toxicity–along with the cellular pathways needed for tolerance–remain unclear. In this study, we conducted a genome-wide functional screen in *S. cerevisiae* to identify nonessential deletion mutants displaying altered growth in the presence of FA, with the goal of more clearly defining the cellular processes required for FA tolerance. Herein we report that yeast genes required for FA tolerance revealed in this study include those implicated in various DNA repair pathways, RNA turnover, osmoregulation, and stress response, with many conserved in humans (Table 2).

**Table 2:** **Human orthologs of yeast genes confirmed as required for FA tolerance**.

**Yeast gene**	**Human ortholog(s)**	**Human protein description**
*GLO3*	*ARFGAP2*	GTPase-activating protein implicated Golgi/ER transport
*HOG1*	Various *MAPKs*	Involved in various MAP kinase signal transduction pathways
*MRE11*	*MRE11A*	Component of MRN complex involved in DSB repair
*MUS81*	*MUS81*	Crossover junction endonuclease
*RAD1*	*ERCC4 (XPF)*	Endonuclease responsible for 5′-incision during nucleotide excision repair
*RAD5*	*HLTF*	Helicase/ubiquitin ligase; plays role in error-free postreplication DNA repair
*RAD10*	*ERCC1*	Component of endonuclease responsible for 5'-incision during DNA repair
*RAD18*	*RAD18*	E3 ubiquitin-protein ligase involved in postreplication repair of damaged DNA
*RAD27*	*FEN1*	Removes 5′ overhanging flaps in DNA repair
*RAD50*	*RAD50*	Component of MRN complex involved in DSB repair
*RAD51*	*RAD51*	Involved in homologous recombination and DSB repair
*RAD57*	*XRCC3*	Involved in homologous recombination repair pathway of dsDNA
*RAD59*	*RAD52*	Involved in DSB repair
*SFA1*	*ADH5*	Catalyzes oxidation of long-chain alcohols and S-(hydroxymethyl) glutathione
*SGS1*	*WRN/BLM*	Participates in DNA replication and repair
*SKI2*	*SKIV2L*	Associated with RNA exosome; may be involved in pre-mRNA splicing
*SKI3*	*TTC37*	Component of SKI complex; may be involved in RNA decay
*SKI7*	*HBS1L*	Member of GTP-binding elongation factor family
*SKI8*	*WDR61*	Component of SKI complex; may be involved in RNA decay
*SSK2*	*MAP3K4*	Component of protein kinase signal transduction cascade

*Deletion of the yeast genes listed resulted in sensitivity to FA in this study (shown in alphabetical order), and the human orthologs of these genes are displayed*.

### Links to prior investigations of formaldehyde in *saccharomyces cerevisiae*

Although yeast cannot be used as model to assess carcinogenicity, its favorable characteristics for toxicological studies have led several groups to utilize the organism to assess FA toxicity. In general, the investigations by other groups contrast with ours in that we employed quantitative and sensitive measures of toxicity (i.e., growth inhibition) at doses closer to environmental exposure (0.6 mM or less), considering endogenous levels of FA in human blood and tissue range from approximately 0.08–0.4 mM (Andersen et al., 2010; Heck and Casanova, 2004; National Toxicology Program (NTP), 2010). Initial studies of FA toxicity in yeast found high concentrations of FA (17–83 mM) increase recombination (Chanet et al., 1975), alter excision repair mutant survival (Chanet et al., 1976), and generate DNA-protein crosslinks (Magana-Schwencke and Ekert, 1978). Although these analyses utilized much higher doses, the results are generally congruent to ours. More recently, Grogan and Jinks-Robertson (2012) demonstrated FA-generated DNA lesions can trigger error-prone translesion synthesis (TLS; a subset of PRR) and are substrates for the NER pathway. Lending support to these results, we demonstrate PRR mutants are sensitive to FA (Figure 8); however, we did not identify NER as required following FA treatment. Others have utilized genomic tools to assess FA toxicity in yeast, including Yasokawa et al. (2010), who examined gene expression via microarrays following exposure to 1.8 mM FA, finding metabolism and cell rescue (including DNA repair) genes were up-regulated, whereas protein synthesis genes were down-regulated. In a study comparable to ours, but using higher doses in solid media, de Graaf et al. (2009) performed a screen of the yeast deletion collection to identify mutants affected by acute (60 mM) and chronic (1.5 mM) FA exposure. This survey found DNA repair was similarly required for FA tolerance (i.e., DNA repair mutants were sensitive to FA), with the authors noting that homologous recombination was important for survival at lower doses (1.5 mM)–consistent with our results–while NER was important at high doses (60 mM).

### Links to prior genomics investigations of formaldehyde in other model organisms

Microarrays and other genomics methods have also been utilized in various models to explore potential mechanisms of FA toxicity. Andersen et al. (2008) assessed gene expression in rats exposed to FA, finding genes associated with DNA repair showed a transcriptomic response to FA. However, these results were only demonstrated at high doses, and the induction of DNA repair genes was not observed at doses lower than those that induce tumors in rodent bioassays. DNA damage response and/or DNA repair gene expression was not altered in additional FA gene expression studies in rats and humans (Sul et al., 2007; Neuss et al., 2010; Zeller et al., 2011), but Giaever et al. (2002) has demonstrated a gene's expression is generally unrelated to its requirement for growth in a certain condition. In another study similar to ours–in which gene deletion mutants were used to examine FA toxicity–Ridpath et al. (2007) utilized a panel of avian DT40 knockout cell lines to show that cells deficient in homologous recombination and translesion synthesis, but not NHEJ, were hypersensitive to FA treatment, results strikingly analogous to those we report herein. Moreover, Shen et al. (2016) screened a human haploid cell mutant library to identify and validate six mutants resistant to FA, including those lacking genes involved in amino acid metabolism, the urea and tricarboxylic acid cycles, the progression of meiosis, telomere replication, immunoglobulin production, and MAPK signaling.

### RNA turnover and formaldehyde tolerance

Our data indicate the conserved SKI complex, which mediates RNA degradation by the exosome (Brown et al., 2000), is required for FA tolerance (Figure 3). To our knowledge, this is the first time SKI has been linked to FA toxicity, as neither prior functional yeast data (de Graaf et al., 2009) or queries at the Comparative Toxicogenomics Database (Davis et al., 2016) revealed functional or gene expression associations, respectively, between SKI components and FA in any organism. RNA degradation, ubiquitous in all cells, is fundamentally linked to RNA processing, turnover, and surveillance; accordingly, it is an important homeostatic regulator (reviewed by Houseley and Tollervey, 2009). SKI participates in many cytoplasmic pathways of the exosome complex, a conserved nuclease that degrades RNAs in the 3′-to-5′ direction, including those involved in routine turnover of normal mRNAs, and the degradation of aberrant mRNAs (i.e., those with defects in processing, folding, or assembly with proteins) via nonsense-mediated decay and non-stop mRNA decay (reviewed by Halbach et al., 2013). Mutations in human SKI complex subunits cause syndromic diarrhea/trichohepatoenteric syndrome (Fabre et al., 2013).

Considering FA can crosslink macromolecules such as proteins and DNA–incidentally, these properties are widely utilized to detect and quantify molecular interactions (reviewed by Hoffman et al., 2015)–one explanation for the FA sensitivity of SKI mutants is as follows: FA may produce adducts on RNA and/or promote the formation of RNA-RNA, RNA-DNA, or RNA-protein crosslinks, and without a fully functional SKI, aberrant RNA molecules are not properly or efficiently degraded. The subsequent accumulation of defective messenger, transfer, ribosomal, regulatory, or other RNA molecules may overwhelm the cell by multiple mechanisms, possibly by disrupting DNA replication through formation of RNA-DNA hybrids or causing the sequestration of RNA-binding proteins (for a review, see Houseley and Tollervey, 2009).

### DNA damage response and formaldehyde tolerance

DNA-protein crosslinks (DPCs) are thought to play a significant role in FA-mediated genotoxicity and carcinogenicity, as DPCs have been detected *in vitro* and *in vivo* in both humans and animals, and the induction of DPCs by FA is dose-dependent and correlates with tumorigenesis (National Toxicology Program (NTP), 2010). Our functional profiling data demonstrate that some form of DNA damage is a major mechanism of FA toxicity in yeast, and these results are generally consistent with a requirement for DPC tolerance and repair. These results may extend to or provide data for additional toxicological studies with other structurally related small aldehydes, such as acetaldehyde. This chemical—which also causes DPCs both *in vitro* and *in vivo*—is of concern due to widespread exposure from natural and industrial sources, and has been classified by IARC as a human carcinogen, as associated with consumption of alcoholic beverages (IARC, 2012b).

DPCs can be repaired by various mechanisms; if the lesion cannot be removed by NER or base excision repair (BER), the replication fork may arrest at the site of damage, eliciting HR and/or damage tolerance systems such as PRR/TLS to help restart the stalled replication fork (Grogan and Jinks-Robertson, 2012; Stingele and Jentsch, 2015). Nakano et al. (2007) demonstrated that in bacteria, NER repairs DPCs with small crosslinked proteins, whereas RecBCD-dependent HR processes oversized DPCs. The same group found HR, not NER, is the major contributor to DPC tolerance in mammalian cells, while also showing DPCs accumulate in HR-deficient cells–suggesting fork breakage at DPCs initiates HR to reactivate stalled forks (Nakano et al., 2009). The Fanconi anemia pathway may mediate HR repair of DPCs in higher eukaryotes (Stingele and Jentsch, 2015); Ren et al. (2013) has illustrated human lymphoblasts deficient in *FANCD2*, a homologous repair gene involved in DNA crosslink repair via the Fanconi anemia pathway, were more susceptible to FA, with DPCs increasing in a dose-dependent manner. While there are a lack of Fanconi anemia functional homologs in yeast, our studies confirmed a requirement for *RAD5, RAD18*, and *RAD51*, genes identified as factors in the Fanconi-like crosslink pathway in yeast (Daee and Myung, 2012), in FA tolerance—suggesting HR repair may be mediated by the Fanconi-like pathway in response to FA in yeast. Taken together, these data, combined with congruent results in yeast (de Graaf et al., 2009) and avian cells (Ridpath et al., 2007), indicate HR plays a pivotal role in the repair of FA-induced DPCs in more complex organisms.

Previous work suggests that NER plays a role in FA tolerance. In a test of various NER-deficient Chinese Hamster Ovary (CHO) cell lines, XPF (*RAD1* in yeast) and ERCC1 (*RAD10* in yeast) deficient cells were the most sensitive to FA (Kumari et al., 2012). However, HR deficient cells were not tested, and the authors propose NER may process secondary lesions generated during DPC repair (i.e., DPCs are converted to single-strand or double-strand breaks that must be repaired by NER). This hypothesis is strengthened by the findings of de Graaf et al. (2009), where single-strand break formation following acute FA exposure in yeast was observed as NER-dependent. We similarly demonstrate that deletions in the yeast NER genes *RAD1* or *RAD10* result in FA sensitivity (Figure 7), suggesting that these proteins may perform analogous functions in yeast, although additional NER genes were not identified by our screen.

Taken together, our results support and help clarify the proposed mechanism of DNA damage and repair broadly outlined by Ridpath et al. (2007): first, FA induces DPCs. If the cell is replicating, DPCs may cause stalled replication forks, which may be addressed by HR and result in error-free repair. If the cell is not replicating, DPCs may be degraded to DNA-amino acid crosslinks (DACs) that are repaired by NER, again resulting in error-free repair. If NER pathways are saturated or the cell begins replicating before DACs are repaired by NER, HR or error-prone PRR (TLS) pathways may act to bypass the damage. However, if HR pathways are also saturated, then the cell may suffer chromosomal aberrations. FA thus likely mediates DNA damage through multiple mechanisms dependent on dose and coincident cellular stressors.

## Conclusions

We have used functional toxicogenomics to identify yeast deletion strains susceptible to treatment with FA, a human carcinogen and potential leukemogen. This study has demonstrated the importance of multiple conserved DNA repair pathways in FA tolerance in yeast and has identified other conserved genes (e.g., the SKI complex) not previously implicated in FA toxicity. Individuals with deficiencies in DNA repair or RNA turnover may be more susceptible to FA. This study highlights *S. cerevisiae* as an effective model for identifying cellular pathways required for toxicant tolerance as well as potential biomarkers of toxicant susceptibility.

## Author contributions

MN, CV, LZ, and MS conceived and designed the experiments, while MN and CR performed the experiments. Data was analyzed by MN, BG, VD, AL, LZ, and CV. The manuscript was prepared by BG, MN, VD, CV, and LZ.

### Conflict of interest statement

The authors declare that the research was conducted in the absence of any commercial or financial relationships that could be construed as a potential conflict of interest. The reviewer SA and handling Editor declared their shared affiliation, and the handling Editor states that the process nevertheless met the standards of a fair and objective review.

## Supplementary material

Figure S1 **Network mapping identifies additional DNA-related processes required for FA tolerance**. Cytoscape analyses were performed as indicated in Figure 2. Genetic subnetworks for selected GO categories are shown, where node color (green to white) corresponds to strain fitness score and edge indicates the type of interaction (physical/genetic) between the genes.

Figure S2 **Mutants deficient in formaldehyde metabolism are sensitive to FA**. The AUC was calculated for strains treated with FA and expressed as a percentage of the AUC for the untreated strain. All bars represent the mean and SE for three independent cultures. Statistical significance between the wild-type and mutant strains was calculated with Student's *t*-test, where ^***^*p* < 0.001, ^**^*p* < 0.01, and ^*^*p* < 0.05.

Table S1 **Mutants displaying altered growth in FA**. DSSA identified strains as sensitive or resistant to FA, with negative and positive log2 values indicating sensitivity and resistance, respectively, at the corresponding concentrations.

Table S2 **Overrepresented GO categories within Cytoscape network data**. Output from the Cytoscape plugin BiNGO is shown with a *p*-value cutoff of 0.001. See Figures 2 and S1 for pictorial representations of these data.
