# Supplementary figures and images for: Functional Toxicogenomic Profiling Expands Insight into Modulators of Formaldehyde Toxicity in Yeast
Source: Front Genet. 2016 Nov 17;7:200. doi: 10.3389/fgene.2016.00200 (PMC5112362; doi:10.3389/fgene.2016.00200)

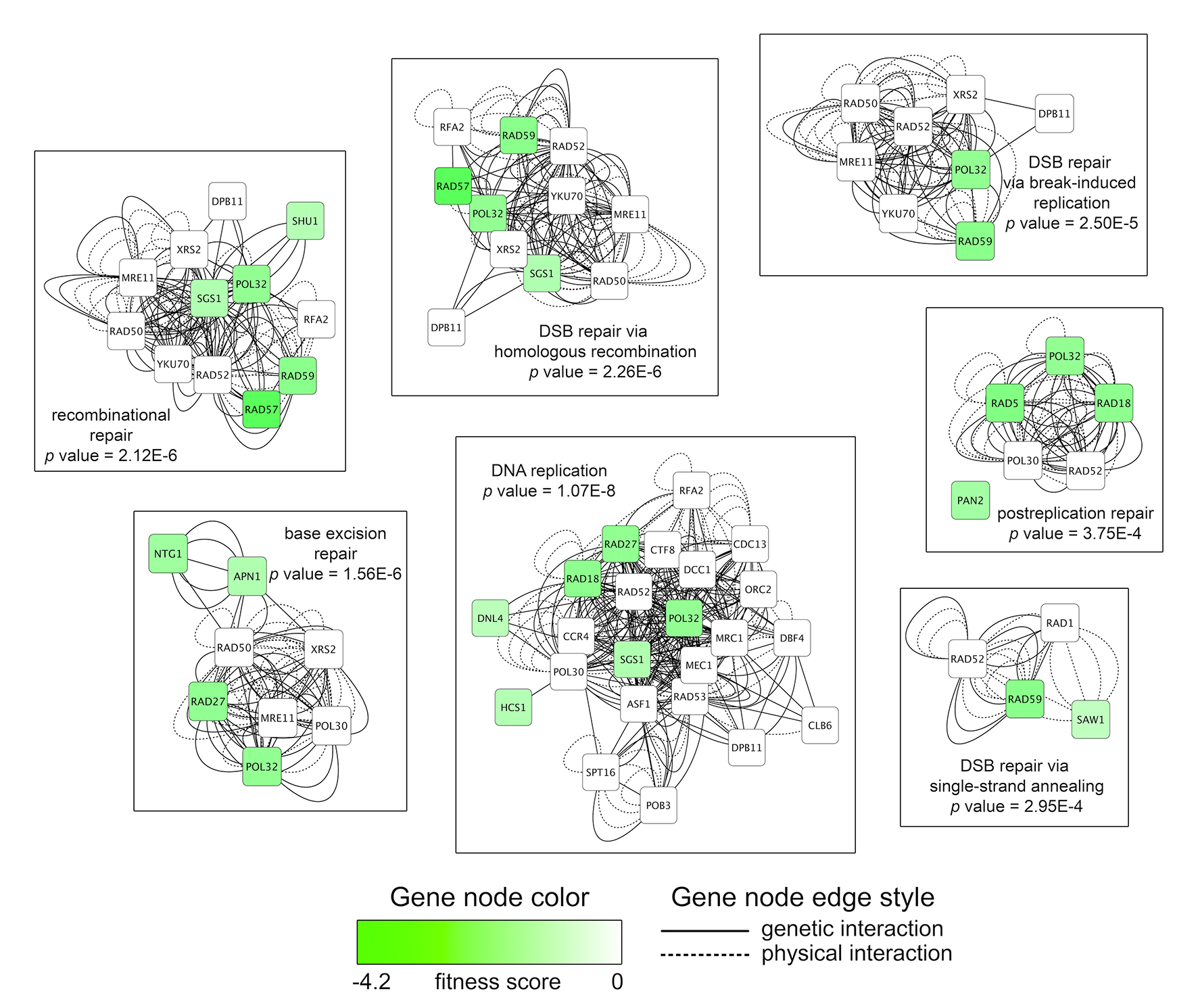

Supplement: Figure S1 — Network mapping identifies additional DNA-related processes required for FA tolerance. Cytoscape analyses were performed as indicated in Figure 2. Genetic subnetworks for selected GO categories are shown, where node color (green to white) corresponds to strain fitness score and edge indicates the type of interaction (physical/genetic) between the genes. [file Image1.TIF]

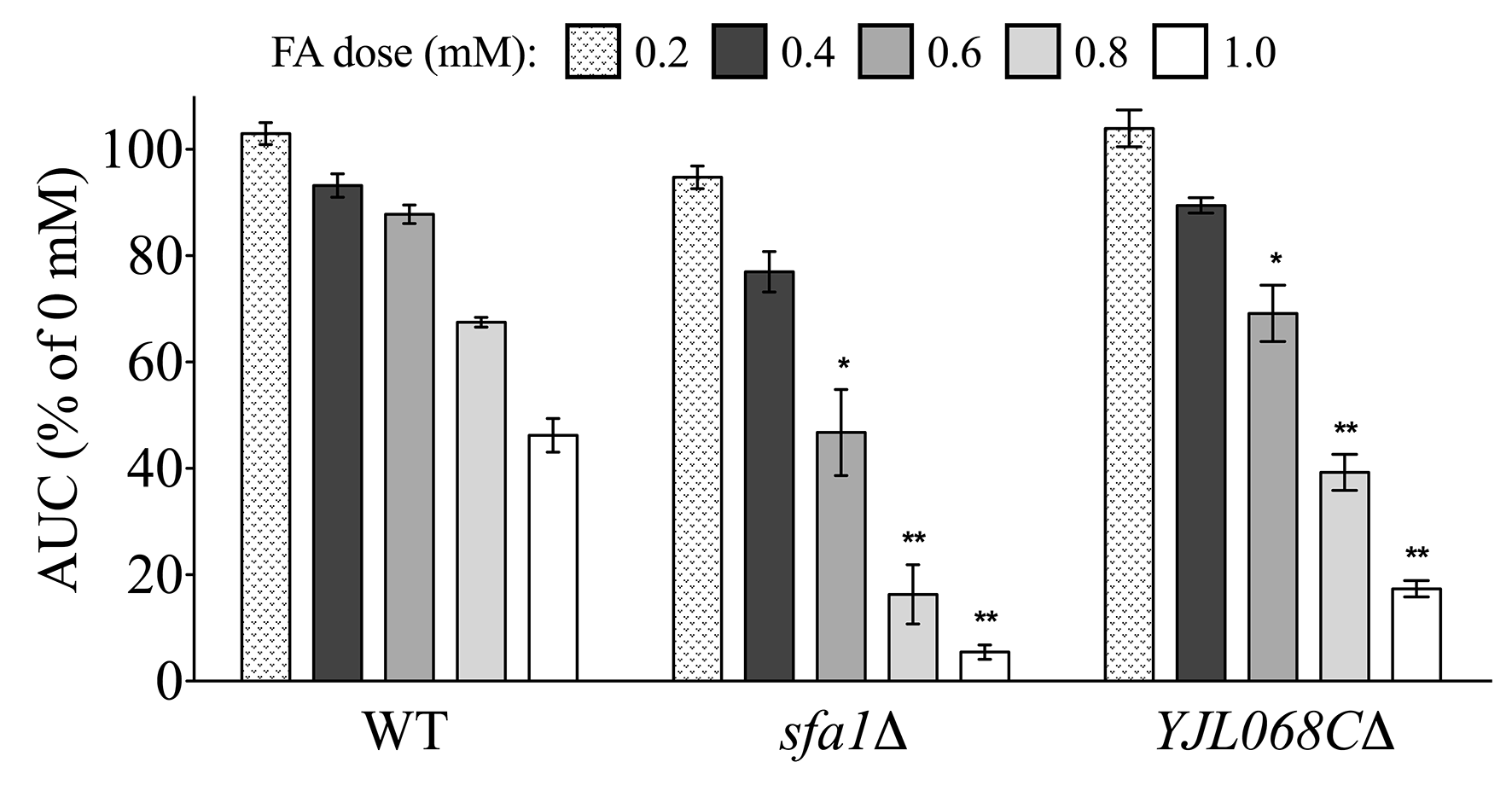

Supplement: Figure S2 — Mutants deficient in formaldehyde metabolism are sensitive to FA. The AUC was calculated for strains treated with FA and expressed as a percentage of the AUC for the untreated strain. All bars represent the mean and SE for three independent cultures. Statistical significance between the wild-type and mutant strains was calculated with Student's t-test, where ***p < 0.001, **p < 0.01, and *p < 0.05. [file Image2.tiff]
